# Acetylsalicylic acid (Aspirin®) and liver regeneration: experimental study in rats

**DOI:** 10.1590/0100-6991e-20213164

**Published:** 2021-11-05

**Authors:** MARIA DE LOURDES PESSOLE BIONDO-SIMÕES, VICTOR CEZAR DE AZEVEDO PESSINI, CAROLINA AYUMI ICHI, ROGÉRIO RIBEIRO ROBES, SÉRGIO IOSHII

**Affiliations:** 1 - Universidade Federal do Paraná (UFPR), Departamento de Cirurgia - Curitiba - PR - Brasil; 2 - Universidade Federal do Paraná (UFPR) - Curitiba - PR - Brasil; 3 - Universidade Federal do Paraná (UFPR) - Curitiba - PR - Brasil; 4 - Universidade Federal do Paraná (UFPR), Departamento de Veterinária - Curitiba - PR - Brasil; 5 - Universidade Federal do Paraná (UFPR), Departamento de Anatomia Patológica - Curitiba - PR - Brasil

**Keywords:** Liver Regeneration, Aspirin, Hepatectomy, Regeneração Hepática, Aspirina, Hepatectomia

## Abstract

**Objective:**

to evaluate the influence of acetylsalicylic acid (ASA) on cell proliferation after partial hepatectomy in rats.

**Methods:**

40 male Wistar rats were separated into four groups of ten rats each. Groups 1 and 2 (controls): undergoing 30% partial hepatectomy and, after one day (group 1) and seven days (group 2), to euthanasia; daily administration of 0.9% saline solution (1mL per 200g of body weight). Groups 3 and 4 (experimental): undergoing 30% partial hepatectomy and, after one day (group 3) and seven days (group 4), to euthanasia; daily administration of ASA (40mg/mL, 1mL per 200g of body weight). The absolute number of cells stained with PCNA was counted in photomicrographs, in five fields, and it was calculated the mean of positive cells per animal and per group.

**Results:**

the final mean of PCNA+ cells per group was: in group 1, 17.57 ± 6.77; in group 2, 19.31 ± 5.30; in group 3, 27.46 ± 11.55; and, in group 4, 12.40 ± 5.23. There was no significant difference at the two evaluation times in the control group (p=0.491), but there was in the experimental group (p=0.020), with a lower number of PCNA+ cells on the seventh day. The comparison between the two groups, on the first day, showed more PCNA+ cells in the livers of the animals that received ASA (p=0.047), and on the seventh day the number was lower in the experimental group (p=0.007).

**Conclusion:**

ASA induced greater hepatocyte proliferation.

## RESUMO

**Objetivo:**

avaliar a influência do ácido acetilsalicílico (AAS) na proliferação celular após hepatectomia parcial em ratos.

**Métodos:**

40 ratos Wistar machos foram separados em quatro grupos com dez ratos cada. Grupos 1 e 2 (controles): submetidos à hepatectomia parcial de 30% e, após um (grupo 1) e sete dias (grupo 2), à eutanásia; administração diária de solução fisiológica 0,9% (1mL por 200g de peso). Grupos 3 e 4 (experimentos): submetidos à hepatectomia parcial de 30% e, após um (grupo 3) e sete dias (grupo 4), à eutanásia; administração diária de AAS (40mg/mL, 1mL por 200g de peso). Realizou-se a contagem do número absoluto de células coradas com PCNA em fotomicrografias, em cinco campos e cálculo da média de células positivas por animal e por grupo.

**Resultados:**

A média final de células PCNA+ por grupo foi: no grupo 1, de 17,57 ± 6,77; no grupo 2 de 19,31 ± 5,30; no grupo 3, de 27,46 ± 11,55; e, no grupo 4, de 12,40 ± 5,23. Não houve diferença significante nos dois tempos de avaliação no grupo controle (p=0,491), mas houve no grupo experimento (p=0,020), observando-se menor número de células PCNA+ no sétimo dia. A comparação entre os dois grupos, no primeiro dia, mostrou mais células PCNA+ nos fígados dos animais que receberam AAS (p=0,047), e no sétimo dia o número foi menor no grupo experimento (p=0,007).

**Conclusão:**

O AAS induziu maior proliferação hepatocitária.

## INTRODUCTION

One of the main characteristics of the liver is its ability to regenerate. However, the primary response of the organ to different lesions is not always this, and it may concur with fibrosis and hepatocyte necrosis^1^. Hepatic fibrosis results from a sustained response to chronic liver injuries and thrombosis of intrahepatic vessels, which stimulate the exacerbated secretion of the extracellular matrix by stellate cells^2^. Necrosis occurs in contexts of severe hypoxemic or toxic lesions, situations in which liver cells, unable to maintain their basic homeostatic functions, lose their integrity^3^.

The liver has a unique ability to regenerate. Even after removing 70% of its total mass, the remaining tissue is able to regenerate, recovering its original volume and function^4^. This process depends on a series of cytokines and growth factors, which stimulate angiogenesis and coordinate hepatocyte hypertrophy and hyperplasia. 

Platelets seem to play a dual role in liver regeneration: they release pro and anti-fibrotic cytokines, which can either exacerbate parenchymal fibrosis and impair regeneration, or suppress this fibrogenesis and stimulate the regenerative process^5^. Furthermore, the formation of microthrombi in the hepatic venous system mediated by platelet aggregation is associated with liver atrophy and fibrosis in transplanted livers^6^. Therefore, it is suggested that anti-thrombogenic therapies may exert some influence on the reduction of liver fibrosis in these cases^7^.

Acetylsalicylic acid (ASA), like other antiplatelet drugs, seems to play an important role in the modulation of liver fibrosis^7^. ASA is an anti-inflammatory medication with antiplatelet aggregation properties widely used in clinical practice. Its main mechanism of action is the inhibition of the enzyme cyclooxygenase (COX), which converts arachidonic acid into prostaglandins^8^, substrates for the synthesis of thromboxane A2, a compound produced by platelets and responsible for platelet aggregation^9^.

The influence of ASA on the progression of liver fibrosis and its role on the progression of chronic liver diseases are widely discussed topics in the literature. Studies show that treatment with ASA may be associated with a lower risk of progression of liver fibrosis, a reduction in the risk of developing hepatocarcinoma and mortality from chronic liver diseases^10^ ^-^ ^13^.

Most studies address the use of the medication on the progression of fibrosis in already cirrhotic livers^14^ ^-^ ^17^, and the role of ASA is poorly understood in liver regeneration in healthy livers. Thus, the present study aims to evaluate the influence of acetylsalicylic acid administration on liver regeneration in a model of partial hepatectomy in healthy male Wistar rats (non-cirrhotic), taking into account its anti-inflammatory and anti-platelet aggregation effects. 

## METHODS

The project was submitted to the Ethics Committee for the Use of Animals of the Biological Sciences Sector of the Federal University of Paraná, which was approved and received registration 23075.045830/2018-25, under protocol number 1214.

The sample consisted of 40 male Wistar rats (*Rattus norvegicus albinus, Rodentia mammalia*), aged between 100 and 120 days and weighing between 300 grams and 480 grams, with a mean of 399 ± 53.84 grams. The animals were housed in the Laboratory of the Discipline of Surgical Technique and Experimental Surgery of the Federal University of Paraná, where they remained during the quarantine period and throughout the experiment. The sample size was calculated based on previous works with a similar research protocol approved by CEUA-BIO^18^ ^,^ ^19^. 

The temperature was maintained at 20 ± 2 degrees centigrades, the air changes, the characteristic of the environment and the luminosity according to 12-hour light and dark cycles. The animals were kept in polypropylene boxes, appropriate for the species, containing white shavings (changed daily), in groups of five animals per box. They received water and standard commercial food, suitable for the species, ad libitum.

The sample was randomly divided into four groups, with ten rats each. The animals in groups 1 and 2 constituted the controls and those in groups 3 and 4, the experiments. All underwent partial hepatectomy. The animals in the control groups received daily sodium chloride solution, 0.9%, 1mL per 200g of body weight, by gavage, and those in the experimental groups received acetylsalicylic acid 40mg/mL, 1mL per 200g of body weight^20^. The animals in groups 1 and 3 were euthanized 24 hours after the intervention and those in groups 2 and 4 on the seventh day after the intervention. The medication was started one day before the intervention and was maintained until euthanasia.

Partial hepatectomy was performed according to the modified method by Higgins and Anderson (1931)^21^, with resection of approximately 30% of the liver. Anesthesia was performed by a veterinarian-anesthetist, with intramuscular injection of ketamine hydrochloride (50mg/kg) and xylazine hydrochloride (20mg/kg), complemented with induction via inhalation with isoflurane 1 to 1.5% under mask, associated with 100% of oxygen. Trichotomy of the ventral abdominal wall was performed, antisepsis was carried out with polyvinylpyrrolidone-iodine (PVP-I) and a median incision of four centimeters. The ligamentum teres hepatis was sectioned, isolating the left lateral lobe, which, after ligation with 4.0 cotton thread, was resected. After the review of hemostasis, laparorrhaphy was performed in two planes, the first, the peritoneum-muscle-aponeurotic plane and the second, the skin, with synthesis in continuous running with a 4.0 nylon monofilament thread. Intramuscular dipyrone (10mg/kg) was used for analgesia. 

After the period determined for each group, euthanasia was performed under anesthesia, according to the protocol described in the Guidelines for the Practice of Euthanasia of the National Council for the Control of Animal Experimentation (2013)^22^ and the Brazilian Guide for Good Practices in Euthanasia in Animals of the Federal Council of Veterinary Medicine (2013). Anesthetic induction was performed with inhaled isoflurane and thiopental sodium solution (10mg/kg) was administered intravenously, followed by cardiac puncture for administration of 10% potassium chloride solution (5mg/kg).

Collection of the remaining liver started with trichotomy and antisepsis of the abdominal wall. This was followed by the opening of the cavity with a “U” incision, which, when folded cranially, allowed for the exposure and total resection of the organ.

The dried pieces were fixed in 10% buffered formalin, sending them for histological analysis. Four-micrometer-thick sections were prepared for staining with immunohistochemistry with primary monoclonal proliferating cell nuclear antigen (PCNA) antibody using the strepto-avidin-biotin-peroxidase technique.

**Figure 1:**
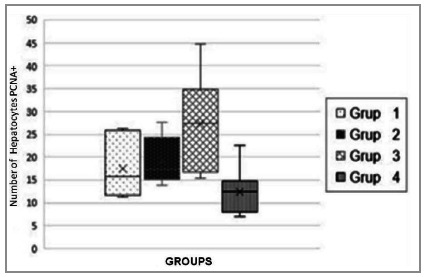
Histological section photomicrograph of liver demonstrating PCNA+ cells.

Legend: Group 1: one day control group (0.9% saline solution; 1mL per 200g of body weight). Group 2: seven days control group (0.9% saline solution; 1mL per 200g of body weight). Group 3: one day experimental group (ASA 40mg/mL; 1mL per 200g of body weight). Group 4: seven days experimental group (ASA 40mg/mL; 1mL per 200g of body weight).

The analysis of cell proliferation was based on counting the absolute number of cells labeled by anti-PCNA (PCNA+ cells) in photomicrographs of histological sections and subsequent calculation of the mean number of positive cells per animal and per group (Figure 1). The ZEN Blue software (Carl Zeiss Microimaging, Jena, Germany) was used for cell counting. For each animal, four slides were made and, on each slide, five fields with an area of 4.84cm², randomly marked, were analyzed at 400x magnification. The counting was carried out by two independent observers and, if there was an interobserver difference greater than 30% between the final means obtained for the group, the analysis was carried out by a third observer.

The collected data underwent statistical analysis using two-tailed non-parametric Mann-Whitney tests for comparison between groups 1 versus 3 and 2 versus 4; and Kruskal-Wallis for comparison between groups 1 versus 2 and 3 versus 4, adopting p<0.05 (5%) as the level of significance.

## RESULTS

There were two deaths in groups 2 and 4 and three deaths in groups 1 and 3. One animal in group 1 and one in group 3 were excluded from the statistical analysis due to a technique artifact in the histological preparation. The final sample consisted of 28 animals, distributed as follows: group 1 (six animals), group 2 (eight animals), group 3 (six animals), group 4 (eight animals).

**Figure 2:**
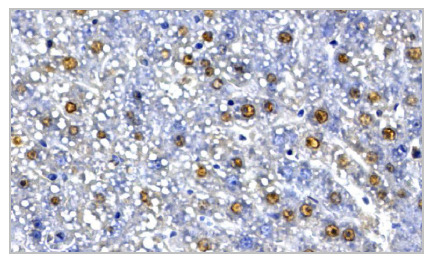
Legend: Immunohistochemistry, 400X. PCNA+ cells stained brown.

The mean of PCNA+ cells between all groups ranged from 7.10 to 44.70, with a total mean of 18.60 ± 8.74. The mean of PCNA+ cells per animal ranged, within group 1, between 11.32 and 26.20, with a final mean of the group of 17.57 ± 6.77; within group 2, between 13.90 and 27.67, with a mean of 19.31 ± 5.30; within group 3, between 15.28 and 44.70, with a mean of 27.46 ± 11.55; and, within group 4, between 7.10 and 22.60, with a mean of 12.40 ± 5.23 (Table 1; Figure 2).

### Appendix

**Table 1:** PCNA + Hepatocytes.

	Groups			
	1	2	3	4
	14.67	23.60	21.47	7.70
	11.32	17.40	15.28	7.10
	25.85	14.30	44.70	8.20
	11.60	24.97	16.80	16.67
	15.75	16.55	34.80	11.40
	26.20	16.07	31.70	22.60
		27.67		12.43
		13.90		13.10
Mean	17.57	19.31	27.46	12.40
Standard Deviation	6.77	5.30	11.55	5.23
% of Standard Deviation	38.53	27.45	44.03	42.78
Maximum	26.20	27.67	44.70	22.60
Minimum	11.32	13.90	15.28	7.10
1 x 2	Kruskal-Wallis Test	p=0.439		
3 x 4	Kruskal-Wallis Test			
1 x 3	Mann-Whitney Test	p=0.047		
2 x 4	Mann-Whitney Test	p=0.007		

Legend: Group 1: one day control group (0.9% saline solution; 1mL per 200g of body weight). Group 2: seven days control group (0.9% saline solution; 1mL per 200g of body weight). Group 3: one day experimental group (ASA 40mg/mL; 1mL per 200g of body weight). Group 4: seven days experimental group (ASA 40mg/mL; 1mL per 200g of body weight).

In the intra-group comparison, there was no significant difference when comparing the control groups (1 and 2) (p=0.439), although a greater number of PCNA+ cells was found on the seventh day (1 day = 17.57 ± 6.77 and 7 days = 19.31 ± 5.30). In the experimental group (treated with ASA) there was a higher number of cells on the first day and a lower number after seven days (p=0.020) (1 day = 26.78 ± 12.08 and 7 days = 12.40 ± 5.23).

In the inter-group comparison, it could be verified that there was a greater number of PCNA+ cells in the experimental group (ASA) in the one-day evaluation (Group 1 = 17.57 ± 6.77 and Group 3 = 27.46 ± 11 .55), this difference was significant (p=0.047). The comparison between the two groups, after seven days, showed that there was a greater number of PCNA+ cells in the control group (p=0.007), (Group 2 = 19.31 ± 5.30 and Group 4 = 12.40 ± 5.23).

## DISCUSSION

The partial hepatectomy model in rats is the classic for studying liver regeneration, and it has been used for decades^23^. The model described by Higgins and Anderson (1931)^21^ proposes the surgical removal of two thirds of the liver, resulting in immediate activation of the regenerative process and complete recovery of the organ mass after seven to ten days^24^ ^-^ ^26^. The popularity of the model is based on two important aspects: the resection of liver tissue is not associated with massive necrosis of the remaining tissue and the regeneration time can be precisely measured^27^.

The liver of rats is composed of five lobes: right lobe (38%), left lobe (30%), caudate lobe (8%) and square lobes (10%)^28^. The multilobular structure of the rat liver, with long and individualized vascular hila, allows lobar resection en bloc, leaving a minimum of residual ischemic tissue^28^. In addition, the section of the liver parenchyma immediately stimulates the beginning of regeneration^23^ ^-^ ^27^.

The induction of regeneration occurs at a time when virtually all hepatocytes are quiescent in phase G028; DNA synthesis begins 12-16 hours after hepatectomy and peaks at 24-48 hours^23^ ^,^ ^29^. Thus, the first 24 hours after the procedure provide a single period for studying hepatocytes, which start the cell cycle, going from the G0 to G1 state and from G1 to the S phase in relative synchrony^30^. The regeneration rate then peaks between one and four days (when analyzed in terms of liver weight, liver regeneration rate and hepatocyte proliferation), being completed after eight days^31^. 

In the present study, the impact of acetylsalicylic acid (ASA) on liver regeneration after partial hepatectomy in rats was analyzed. It was decided to modify the technique described by Higgins and Andersen (1931), adopting a single resection of the left hepatic lobe (approximately 30% of the organ volume), due to the high mortality when the pilot study was carried out, and 70% of the liver mass was ressected. Liver regeneration was evaluated one and seven days after the procedure, according to the literature indication that DNA synthesis reaches its peak 24 hours^29^ after resection and that the regeneration process is complete after seven to ten days^24^ ^-^ ^26^ ^,^ ^31^.

There are several methods to quantify liver regeneration, including: liver mass, mitotic cell count, identification of DNA synthesis, and immunohistochemical methods. Among these, staining with proliferating cell nuclear antigen (PCNA) antibody is one of the most common^32^. PCNA is an auxiliary protein of the DNA polymerase delta enzyme, essential for DNA replication in prokaryotic cells. Its expression is cell cycle dependent, being initially detected in the G1 phase, with a peak in the S phase. In this regard, it acts as a marker for the entry of quiescent cells into the cell cycle.

To determine hepatocyte proliferation, the mean number of cells labeled with PCNA (PCNA+) was calculated for each animal and group, and the groups were compared using an appropriate statistical test. In the present study, the mean number of PCNA+ cells among all groups ranged from 7.10 to 44.70, with a total mean of 18.60 ± 8.74. The mean of PCNA+ cells was lower in group 4 (12.40 ± 5.23) and higher in group 3 (27.46 ± 11.55).

The analysis of the first day showed a much higher number of replicating hepatocytes in the ASA group than in the control group (p=0.047). The seven-day evaluation showed a greater number of hepatocytes in replication in the control group (p=0.007). These data demonstrate that, at first, when the regenerative process was installed, animals treated with ASA had a more active replication rate. At seven days, the untreated group showed a greater number of replicating hepatocytes. This observation allows us to imply that the replication process took place earlier and with greater speed in the treated group.

Although Miyaoka et al.^33^ have pointed out cell hypertrophy as the main initial mechanism for increasing liver mass after partial hepatectomy in rats, Marongiu et al.^34^ demonstrated that hepatocyte hyperplasia is the key mechanism in the evolution of liver regeneration, with hypertrophy being only transitory as part of the process. Therefore, in the present study, mass gain and hepatocyte hypertrophy were not evaluated, the effects of ASA on liver regeneration are highlighted only by hepatocyte hyperplasia, with PCNA+ cell count.

Wang et al. demonstrated thrombocytopenia and medication-mediated platelet dysfunction as determining factors in reducing the rate of liver regeneration in rats^35^. Platelets accumulate in the remaining liver tissue within 5 to 15 minutes after partial hepatectomy in rats^36^ ^,^ ^37^ and disappear within an hour of the procedure, which suggests a platelet mitogenic stimulus in the very early stages of the postoperative period^36^.

In humans, there is evidence that postoperative platelet count influences the functionality and regeneration of transplanted liver tissue, as well as the morbidity and mortality of patients undergoing hepatectomy^35^ ^,^ ^38^ ^,^ ^39^. Conversely, thrombocytosis and platelet infusion in the portal vein seem to stimulate liver regeneration after hepatectomy^35^ ^,^ ^37^.

The exact mechanism by which platelets stimulate liver regeneration is still unclear. In vivo studies demonstrate that inflammatory cytokines and growth factors released by platelets may be crucial for the initiation of hepatocyte proliferation^35^ ^,^ ^36^ ^,^ ^40^, and direct contact between platelets and endothelial cells of the hepatic sinusoids seem to play an important role in stimulating to the release of these platelet factors^41^. In vitro studies demonstrate that serotonin, stored in platelets and released at sites of injury as part of the platelet hemostatic action, is a potent mitogenic agent and tissue remodeler^42^ ^,^ ^43^. Thus, the reduction in platelet number or activity after partial hepatectomy could negatively influence the liver regeneration process.

On the other hand, when evaluating the influence of antiplatelet agents in models of liver cirrhosis in rats, Assy et al.^20^ demonstrated that ASA in low doses was able to prevent the progression of fibrosis and to stimulate liver regeneration in cirrhotic livers. Poujol-Robert et al.^14^ observed an association between the use of ASA and a reduction in the progression of fibrosis rates in transplant patients with hepatitis C.

## CONCLUSION

The results of this study demonstrate that the use of ASA stimulated hepatocyte proliferation. 

## References

[1] Wang S, Friedman SL (2020). Hepatic fibrosis a convergent response to liver injury that is reversible. J Hepatol.

[2] Anstee QM, Goldin RD, Wright M, Martinelli A, Cox R, Thursz MR (2008). Coagulation status modulates murine hepatic fibrogenesis Implications for the development of novel therapies. J Thromb Haemost.

[3] Schwabe RF, Luedde T (2018). Apoptosis and necroptosis in the liver a matter of life and death. Nat Rev Gastroenterol Hepatol.

[4] Minuk GY (2003). Hepatic regeneration If it ain't broke, don't fix it. Can J Gastroenterol.

[5] Chauhan A, Adams DH, Watson SP, Lalor PF (2016). Platelets No longer bystanders in liver disease. Hepatology.

[6] Wanless IR, Wong F, Blendis LM, Greig P, Heathcote EJ, Levy G (1995). Hepatic and portal vein thrombosis in cirrhosis possible role in development of parenchymal extinction and portal hypertension. Hepatology.

[7] Li CJ, Yang ZH, Shi XL, Liu DL (2017). Effects of aspirin and enoxaparin in a rat model of liver fibrosis. World J Gastroenterol.

[8] Vane JR (1971). Inhibition of prostaglandin synthesis as a mechanism of action for aspirin-like drugs. Nat New Biol.

[9] Hilário MOE, Terreri MT, Len CA (2006). Antiinflamatórios não-hormonais inibidores da ciclooxigenase 2. J Pediatr.

[10] Iqbal U, Dennis BB, Li AA (2019). Use of anti-platelet agents in the prevention of hepatic fibrosis in patients at risk for chronic liver disease a systematic review and meta-analysis. Hepatol Int.

[11] Lee TY, Hsu YC, Tseng HC (2019). Association of Daily Aspirin Therapy With Risk of Hepatocellular Carcinoma in Patients With Chronic Hepatitis B. JAMA Intern Med.

[12] Hossain MA, Kim DH, Jang JY (2012). Aspirin induces apoptosis in vitro and inhibits tumor growth of human hepatocellular carcinoma cells in a nude mouse xenograft model. Int J Oncol.

[13] Trujillo-Murillo K, Rincón-Sánchez AR, Martínez-Rodríguez H (2008). Acetylsalicylic acid inhibits hepatitis C virus RNA and protein expression through cyclooxygenase 2 signaling pathways. Hepatology.

[14] Poujol-Robert A, Boëlle PY, Conti F (2014). Aspirin may reduce liver fibrosis progression Evidence from a multicenter retrospective study of recurrent hepatitis C after liver transplantation. Clin Res Hepatol Gastroenterol.

[15] Jiang ZG, Feldbrügge L, Tapper EB (2016). Aspirin use is associated with lower indices of liver fibrosis among adults in the United States. Aliment Pharmacol Ther.

[16] Sitia G, Aiolfi R, Di Lucia P (2012). Antiplatelet therapy prevents hepatocellular carcinoma and improves survival in a mouse model of chronic hepatitis B. Proc Natl Acad Sci U S A.

[17] Sahasrabuddhe VV, Gunja MZ, Graubard BI (2012). Nonsteroidal anti-inflammatory drug use, chronic liver disease, and hepatocellular carcinoma. J Natl Cancer Inst.

[18] Biondo-Simões MLP Matias JEF, Martone D Barbos, RF Ogawa GH (2007). Influência da glutamina na regeneração hepática. Revista De Medicina.

[19] Biondo-Simões MLP, Bonato FT, Menacho AM, Drechmer M, Cavalcanti TCS, Felizola SJA (2011). Cicatrização da parede abdominal após hepatectomia parcial. Rev. Col. Bras. Cir.

[20] Assy N, Hussein O, Khalil A (2007). The beneficial effect of aspirin and enoxaparin on fibrosis progression and regenerative activity in a rat model of cirrhosis. Dig Dis Sci.

[21] Higgins G, Anderson G (1931). Experimental pathology of the liver restoration of the liver of the white rat following partial surgical removal. Arch Pathol (Chic).

[22] Concea (2013). Diretrizes da prática de Eutanásia do. Cons Nac Control Exp Anim Resolução Norm.

[23] Forbes SJ, Newsome PN (2016). Liver regeneration - mechanisms and models to clinical application. Nat Rev Gastroenterol Hepatol.

[24] Zafarnia S, Mrugalla A, Rix A (2019). Non-invasive Imaging and Modeling of Liver Regeneration After Partial Hepatectomy. Front Physiol.

[25] Taub R (2004). Liver regeneration from myth to mechanism. Nat Rev Mol Cell Biol.

[26] Nishiyama K, Nakashima H, Ikarashi M (2015). Mouse CD11b+Kupffer Cells Recruited from Bone Marrow Accelerate Liver Regeneration after Partial Hepatectomy. PLoS One.

[27] Michalopoulos GK (2010). Liver regeneration after partial hepatectomy critical analysis of mechanistic dilemmas. Am J Pathol.

[28] Palmes D, Spiegel HU (2004). Animal models of liver regeneration. Biomaterials.

[29] Kountouras J, Boura P, Lygidakis NJ (2001). Liver regeneration after hepatectomy. Hepatogastroenterology.

[30] Diehl AM, Rai R (1996). Review regulation of liver regeneration by pro-inflammatory cytokines. J Gastroenterol Hepatol.

[31] Andersen KJ, Knudsen AR, Kannerup AS (2013). The natural history of liver regeneration in rats description of an animal model for liver regeneration studies. Int J Surg.

[32] Biondo-Simões ML, Matias JE, Montibeller GR, Siqueira LC, Nunes Eda S, Grassi CA (2006). Effect of aging on liver regeneration in rats. Acta Cir Bras.

[33] Miyaoka Y, Ebato K, Kato H, Arakawa S, Shimizu S, Miyajima A (2012). Hypertrophy and unconventional cell division of hepatocytes underlie liver regeneration. Curr Biol.

[34] Marongiu F, Marongiu M, Contini A (2017). Hyperplasia vs hypertrophy in tissue regeneration after extensive liver resection. World J Gastroenterol.

[35] Wang HQ, Yang J, Yang JY, Wang WT, Yan LN (2014). Low immediate postoperative platelet count is associated with hepatic insufficiency after hepatectomy. World J Gastroenterol.

[36] Alkozai EM, Nijsten MW, de Jong KP (2010). Immediate postoperative low platelet count is associated with delayed liver function recovery after partial liver resection. Ann Surg.

[37] Matsuo R, Nakano Y, Ohkohchi N (2011). Platelet administration via the portal vein promotes liver regeneration in rats after 70% hepatectomy. Ann Surg.

[38] Margonis GA, Amini N, Buettner S (2016). Impact of early postoperative platelet count on volumetric liver gain and perioperative outcomes after major liver resection. Br J Surg.

[39] Borowiak M, Garratt AN, Wüstefeld T, Strehle M, Trautwein C, Birchmeier C (2004). Met provides essential signals for liver regeneration. Proc Natl Acad Sci U S A.

[40] Blindenbacher A, Wang X, Langer I, Savino R, Terracciano L, Heim MH (2003). Interleukin 6 is important for survival after partial hepatectomy in mice. Hepatology.

[41] Kawasaki T, Murata S, Takahashi K (2010). Activation of human liver sinusoidal endothelial cell by human platelets induces hepatocyte proliferation. J Hepatol.

[42] Fanburg BL, Lee SL (1997). A new role for an old molecule serotonin as a mitogen. Am J Physiol.

[43] Seuwen K, Pouysségur J (1990). Serotonin as a growth factor. Biochem Pharmacol.

